# A Data-Driven Approach for Fatigue Detection during Running Using Pedobarographic Measurements

**DOI:** 10.1155/2023/7022513

**Published:** 2023-09-26

**Authors:** Zixiang Gao, Liangliang Xiang, Gusztáv Fekete, Julien S. Baker, Zhuqing Mao, Yaodong Gu

**Affiliations:** ^1^Department of Radiology, Ningbo No. 2 Hospital, Ningbo 315010, China; ^2^Faculty of Engineering, University of Pannonia, Veszprém H-8201, Hungary; ^3^Savaria Institute of Technology, Eötvös Loránd University, Szombathely 9700, Hungary; ^4^Auckland Bioengineering Institute, The University of Auckland, Auckland 1010, New Zealand; ^5^Department of Sport and Physical Education, Hong Kong Baptist University, Hong Kong, China; ^6^Faculty of Sports Science, Ningbo University, Ningbo, China

## Abstract

**Background:**

Detecting fatigue at the early stages of a run could aid training programs in making adjustments, thereby reducing the heightened risk of injuries from overuse. The study aimed to investigate the effects of running fatigue on plantar force distribution in the dominant and nondominant feet of amateur runners.

**Methods:**

Thirty amateur runners were recruited for this study. Bilateral time-series plantar forces were employed to facilitate automatic fatigue gait recognition using convolutional neural network (CNN) and CNN-based long short-term memory network (ConvLSTM) models. Plantar force data collection was conducted both before and after a running-induced fatigue protocol using a FootScan force plate. The Keras library in Python 3.8.8 was used to train and tune deep learning models.

**Results:**

The results demonstrated that more mid-forefoot and heel force occurs during bilateral plantar and less midfoot fore force occurs in the dominant limb after fatigue (*p* < 0.001). The time of peak forces was significantly shortened at the midfoot and sum region of the nondominant foot, while it was delayed at the hallux region of the dominant foot (*p* < 0.001). In addition, the ConvLSTM model showed higher performance (Accuracy = 0.867, Sensitivity = 0.874, and Specificity = 0.859) in detecting fatigue gait than CNN (Accuracy = 0.800, Sensitivity = 0.874, and Specificity = 0.718).

**Conclusions:**

The findings of this study could offer empirical data for evaluating risk factors linked to overuse injuries in a single limb, as well as facilitate early detection of fatigued gait.

## 1. Introduction

The popularity of long-distance running as an easily accessible and promoted sport has increased within the last four decades. However, the incidence of musculoskeletal injuries caused by running has also increased rapidly, especially in the lower extremities [1]. The tendons, muscles, or bones of the lower extremities during long-distance running are repeatedly subjected to chronic submaximal loading over a long period of time [2]. As a consequence, overuse injuries are considered to be the most common running injuries [2]. It should, however, be noted that the etiology of running fatigue-induced injuries is multifactorial and complex [3]. Fatigue from long-distance running can shift foot mechanics, potentially causing structural overload [4, 5]. Investigating the relationship between fatigue and the load distribution pattern during running gait has garnered increased interest [6]. A consensus is that an increase in peak metatarsal head pressure occurs after running fatigue [7, 8]. However, inconsistent results have been reported for the influence of running fatigue on the middle foot and heels [2, 7]. Weist et al. [9] demonstrated a significant increase in midfoot pressure and the impulse in the medial heel after performing a running-induced fatigue protocol. Nevertheless, the study by Bisiaux and Moretto [10] found a reduction in pressure and impulse in the midfoot under similar conditions. Willson and Kernozek [11] observed a significant reduction in peak heel pressure after 30 min of high-intensity running.

In addition, more laterally directed roll-off and inadequate pronated heel strikes have been demonstrated to be the potential triggers for lower limb overuse injuries [12]. Previous studies have reported that midfoot, metatarsal, and medial heel loading increases after running-induced plantar muscle fatigue, while loading on the lateral toes decreases [2]. Similar studies have reported that running fatigue causes a reduction in the medial longitudinal arch, which significantly increases mid-toe pressure [13]. In addition, excessive plantar forces of the forefoot lateral were identified as a potential cause for gait-related Achilles tendinopathy [7]. Most of the studies mentioned above did investigate changes in unilateral plantar pressure distribution after fatigue, especially the dominant side. However, there seems to be a lack of empirical data on the effect of running fatigue on the nondominant plantar load distribution. Due to the altered symmetry after fatigue, excessive loading usually occurs in the unilateral limb, especially in the nondominant limb, which may have a weaker fatigue tolerance. Ignoring asymmetric information about bilateral limbs to explore risk factors for fatigue, although it may simplify the data processing and analysis process, may also produce deceptive results. Assuming that changes in bilateral plantar pressure distribution play a primary role in the course of overuse injury development [14, 15], the role of fatigue on both dominant and nondominant plantar distribution should be examined. Therefore, one of the objectives of this study was to determine the effect of running-induced fatigue on bilateral plantar force distribution.

Previous studies have widely shown that fatigue gait risk is associated with shifts in the distribution of bilateral plantar pressure [1, 4, 6, 11]. Coaches and runners can avoid the occurrence of overuse injuries by monitoring fatigue levels in the context of competitive and recreational sports. In addition, excessive fatigue may affect runners' performance and cause secondary injuries to the runner [16]. Therefore, human activity recognition (HAR) methods based on wearable sensors and deep learning algorithms have been widely developed in the last decade [17–19]. Despite significant strides in gait and biomechanics analysis, research into automated fatigue gait recognition with data-driven models remains insufficient [18, 20]. Typical techniques to detect fatigue are surface electromyogram-based collection of muscle activity signals and optical motion capture-based collection of joint kinematics [21, 22]. However, the limited data collection area and the location of the marker attachments make monitoring limited. On the contrary, force plates or insoles with force sensors are easy to use and save time in the experimental setup for data collection. Therefore, this study intends to use a deep learning algorithm based on bilateral plantar pressure data for the early identification of fatigue gait.

Since larger spatial dependencies exist in the pressure data of each plantar region throughout the gait cycle [23, 24], the CNN model has been reported to be better at extracting local spatial features [15]. Similarly, time series data-based plantar pressure data are considered to possess time dependence [25]. However, the plantar pressure distribution data based on time series features may be regarded as static spatial data by the CNN model, and the time-dependent information within the series is lost. Previous studies have shown that long short-term memory network (LSTM) models perform better for the prediction of long-time dependence and nonlinear dynamic changes in a time series [26]. However, LSTM models are less effective in handling spatial relationships of data. The spatial characteristics of the pressure distribution in different plantar regions and the dynamic time characteristics of the variation with time should be considered in the model selection for this study.

The ConvLSTM model will be used in this study on the ground that it transforms the structure of recurrent neural networks into a convolutional structure, thereby preserving the spatial and temporal information of plantar pressure [27]. To verify the performance of the ConvLSTM model for fatigue gait recognition, we used a CNN model to compare the performances. Two hypotheses were proposed: (1) The metatarsal, midfoot, and heel pressures increased in the dominant and nondominant feet after the fatigue intervention, with more significant changes in the nondominant foot; (2) The ConvLSTM model has better performance than the CNN model for automatic recognition of fatigue gait.

## 2. Materials and Methods

### 2.1. Participants

Thirty healthy amateur runners (males) were enlisted from universities and local running clubs for this study. The anthropometric information of the participants is presented in Table 1. The inclusion criteria for this study were that the dominant extremity side was the right extremity side (preferred leg when kicking a ball), the absence of any lower extremity or pelvic musculoskeletal pain in the last 6 months, and running at least 2–3 times per week and for <45 min or <10 km at per running event. The Ethics Committee at Ningbo University approved the study (code: RAGH20210827), and all subjects signed the informed consent.

### 2.2. Data Collection

Subjects were guided by the experimental operator to familiarize the experimental environment (includes ground running tests with barefoot before and after the running-induced fatigue protocol) and process and participated in a 10-min jogging warm-up on a treadmill (Satun h/p/cosmos, Nussdorf–Traunstein, Germany) in advance. A previously identified and validated protocol was employed for building a running-induced fatigue model [28]. With reference to our previously built approach [29], a heart rate sensor band (Polar RS100, United States) and a Borg [30] RPE scale (6–20 scales) were utilized for monitoring fatigue during running. Every participant commenced walking on a treadmill at a velocity of 6 km/h. The pace of gait was augmented by 1 km/h every 2 min until an exertion level of 13 on the Borg scale was attained. Participants sustained the running pace at the established equilibrium until achieving a Borg rating of 17% or 90% of their maximum heart rate (maximum heart rate = 220-age), at which juncture they persisted in running for an extra 2 min. New neutral running shoes were given to every participant for the protocol involving running-induced fatigue.

Pedobarographic data collection was done before and after the running-induced fatigue protocol. Dynamic plantar force data were measured during running using a FootScan pressure plate (size: 2 × 0.4 m, frequency: 480 Hz, RsScan International, Olen, Belgium) embedded in the middle of a 20-m runway. The pressure plate is calibrated using the individual's body weight prior to measurement to avoid errors. Two sets of infrared photocells were placed on either side of the data collection area to monitor the running speed. All participants were required to run barefoot over the data collection area at a speed of 3.3 m/s ±5% [30]. Participants were instructed to use the nondominant foot as the first step on the force plate and to ensure that two consecutive steps were recorded for each trial. Attempts to change the operating mode to strike the pressure plate were ruled out until three valid trial data points were measured before and after the running-induced fatigue protocol.

### 2.3. Data Processing

**Figure 1:**
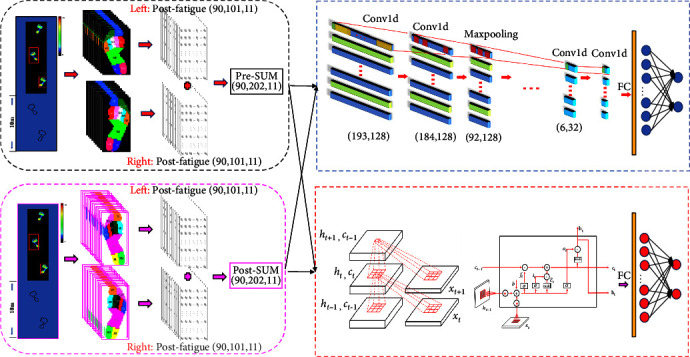
Data collection and analysis process. *Note*. Nondominant side = left foot; dominant side = right foot; H = hallux; OT = other toe; M1-5 = metatarsal 1-5; MF = midfoot; HM = medial heel; HL = lateral heel.

For each trial, 10 plantar anatomical regions were identified by the FootScan application. To avoid recognition errors, the pixels of each area were manually calibrated by an operator. These areas were defined as the hallux (H), other toe (OT), metatarsal 1–5 (M1–M5), midfoot (MF), medial heel (HM), and lateral heel (HL). Time-series attributes of force information for each region and the sum area were interpolated to 101 frames using linear interpolation for statistical comparison. To reduce the effect of individual weight and gait speed differences on the data, all data in this study were annotated using Zavg (total force over the entire support period divided by the total number of frames) [25]. As shown in Figure 1, to preserve asymmetric information of bilateral limbs before and after fatigue, the plantar force data of the nondominant and dominant foot were stitched longitudinally to obtain the bipedal force distribution information of one gait cycle for machine learning training [23].

### 2.4. CNN Model Building

This study uses the Keras Application Programming Interface (API) in Python 3.8.8 for CNN and ConvLSTM model building. CNN models have good performance for feature extraction of input data through convolutional operations of different topological structure kernels. The convolution layer in the model preserves the spatial relationships of the data by using the same convolution operation for each position of the original data. Each type of feature that is extracted generates a feature matrix *Z*. Therefore, after *k* times convolution calculations, the corresponding output matrix *Z*_*k*_ can be represented by Equation (1). In addition, the convolution operation for one-dimensional time series data is also a nonlinear transformation of the original series. Applying a convolution kernel of length *l* to a univariate time series *X* of length *T*, Equation (2) is obtained.

(1) $$\begin{array}{c} Z_{k}=f\left(W_{k}\;*\;X+b\right), \end{array}$$

(2) $$\begin{array}{c} C_{t}=f\left(w\;*\;X_{t-l/2:t+l/2}+b\right)\;|\;\forall t\in \left[1,T\right], \end{array}$$

where *W*_*k*_ and *k* are the convolution kernels (size: *k*_1_ × *k*_2_) and the number of convolution kernels, respectively. *b* is biased, and the convolution operator is defined as ∗. *f* is the activation function that performs a nonlinear transformation in the convolution layers.

**Figure 2:**
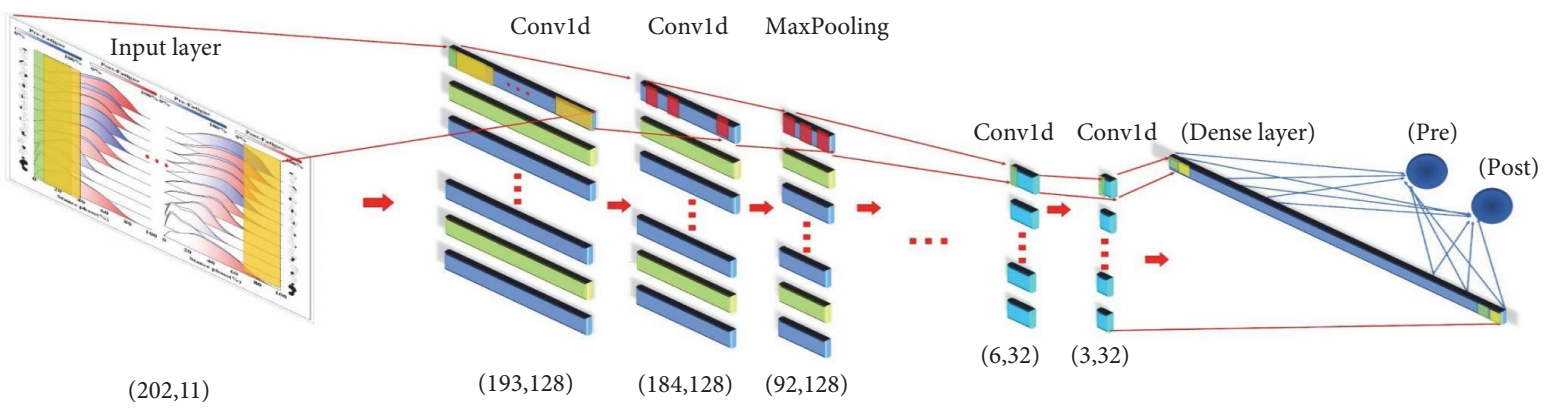
Diagram of the internal structure of the CNN model in this study.

As shown in Figure 2, The optimal CNN model for the recognition of fatigue gait is obtained through repeated debugging parameters. We used a total of eight convolutional layers, three maximum pooling layers, one average pooling layer, one dropout layer, and one dense layer to build the convolutional neural network model. The number of convolution kernels is set to (128, 128, 128, 128, 64, 64, 32, 32). The time step settings are (10, 10, 10, 10, 10, 10, 4, 4). In addition, “RELU” and “Softmax” are set as the activation functions for the convolutional and dense layers, respectively.

### 2.5. ConvLSTM Model Building

**Figure 3:**
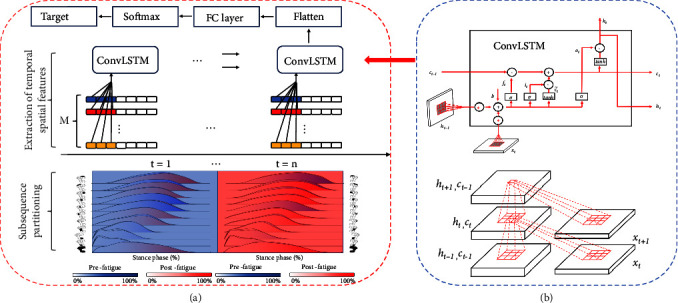
Diagram of the internal structure of the ConvLSTM model in this study, (a) frame diagram; (b) structure diagram.

The convolutional layer extracted the temporal characteristics from the pressure data, while the LSTM layer handled the spatial characteristics (Figure 3(b)). In our ConvLSTM model, operations are depicted by Equations (3)–(8), where ∗ symbolizes the convolution process, and ∘ denotes the Hadamard product.

(3) $$\begin{array}{c} f_{t}=\sigma \left(W_{xf}\;*\;x_{t}+W_{hf}\;*\;h_{t-1}+b_{f}\right), \end{array}$$

(4) $$\begin{array}{c} i_{t}=\sigma \left(W_{xi}\;*\;x_{t}+W_{hi}\;*\;h_{t-1}+b_{i}\right), \end{array}$$

(5) $$\begin{array}{c} {\tilde{c}}_{t}=\tanh\left(W_{xc}\;*\;x_{t}+W_{hc}\;*\;h_{t-1}+b_{c}\right), \end{array}$$

(6) $$\begin{array}{c} c_{t}=f_{t}\circ c_{t-1}+i_{t}\circ {\tilde{c}}_{t}, \end{array}$$

(7) $$\begin{array}{c} o_{t}=\sigma \left(W_{xo}\;*\;x_{t}+W_{ho}\;*\;h_{t-1}+b_{o}\right), \end{array}$$

(8) $$\begin{array}{c} h_{t}=o_{t}\circ \tanh\left(c_{t}\right), \end{array}$$

where *i*_*t*_, *f*_*t*_, and *o*_*t*_ are the input gate, oblivion gate, and output gate, respectively, in the proposed model; *x*_*t*_ represents the data input at the current moment, while *h*_*t*−1_ refers to the output from the hidden layer at the preceding moment. *c*_*t*_ denotes the cell state.

Figure 3(a) shows the framework for building the ConvLSTM model in this research. In this study, we try to make *l* choose a variety of different division lengths, such as 51, 101, 151, 202, and so on, for modeling, and finally find that the model's classification performance is optimal when the subsequence length is *l* = 101. The optimal ConvLSTM for the recognition of fatigue gait is obtained through repeated debugging parameters. We sequentially set up a ConvLSTM layer (number of convolution kernels = 64, kernel size = (1,5)), a dropout layer (random discard ratio = 0.5), a Flatten layer and two dense layers (first: units = 50, activation = RELU”; second: units = 2, activation = “Softmax”) in the final model.

To ensure fast convergence during the training of the binary classification model, the cross-entropy loss function was chosen as the loss function for this study, as shown in Equation (9).

(9) $$\begin{array}{c} L=-\frac{1}{N}\sum_{i=1}^{N}\;\left[y^{\left(i\right)}\log\left({\hat{y}}^{\left(i\right)}\right)+\left(1-y^{\left(i\right)}\right)\log\left(1-{\hat{y}}^{\left(i\right)}\right)\right], \end{array}$$

where *N* is the number of samples and *y*^(*i*)^ and ${\hat{y}}^{\left(i\right)}$ are defined as the true and predicted values.

### 2.6. Statistical Analysis

In this study, a total of 90 cases were sampled, and 80% of the samples were set as the training set and 20% as the test set, where 20% of the training samples were set as the validation set for cross-validation. Therefore, the training set, validation set, and test set samples in this study are 72, 14, and 18. To avoid the occurrence of model underfitting, the number of model iterations was set to 300. This study uses accuracy, sensitivity, and specificity as quantitative metrics for the performance of two classification models. We used fatigue gait as positive samples and normal gait as negative samples. Therefore, Equation (10) was used to assess the overall classification capability of the models. Equations (11) and (12) were used to evaluate the classification capability of negative samples and positive samples of the models, respectively.

(10) $$\begin{array}{c} \text{Accuracy}=\frac{\text{TP}+\text{TN}}{\left(\text{TP}+\text{FN}+\text{FP}+\text{TN}\right)}\times 100\%, \end{array}$$

(11) $$\begin{array}{c} \text{Sensitivity }=\frac{\text{TP}}{\left(\text{TP}+\text{FN}\right)}\times 100\%, \end{array}$$

(12) $$\begin{array}{c} \text{Specificity}=\frac{\text{TN}}{\left(\text{FP}+\text{TN}\right)}\times 100\%, \end{array}$$

where TP and TN are the number of samples correctly identified as fatigue gait and normal gait, respectively, and FP and FN are the number of samples incorrectly identified as fatigue gait and normal gait, respectively. To avoid accidental error, each model is run five times on the test set, and the corresponding classification results are collected.

The Shapiro–Wilk test was performed to check the normality of the data distribution. The paired sample *T*-test of open-source statistical parameter mapping 1d (SPM1d) was used to check the differences between pre- and post-fatigue time-series forces at dominant and nondominant foot. The discrete values of the percentage of time of peak force were checked using a paired sample *T*-test in Python 3.8.8 with the SciPy library. The significance levels were set at 0.05.

## 3. Results

### 3.1. Force Development in Toe and Metatarsal Areas

**Figure 4:**
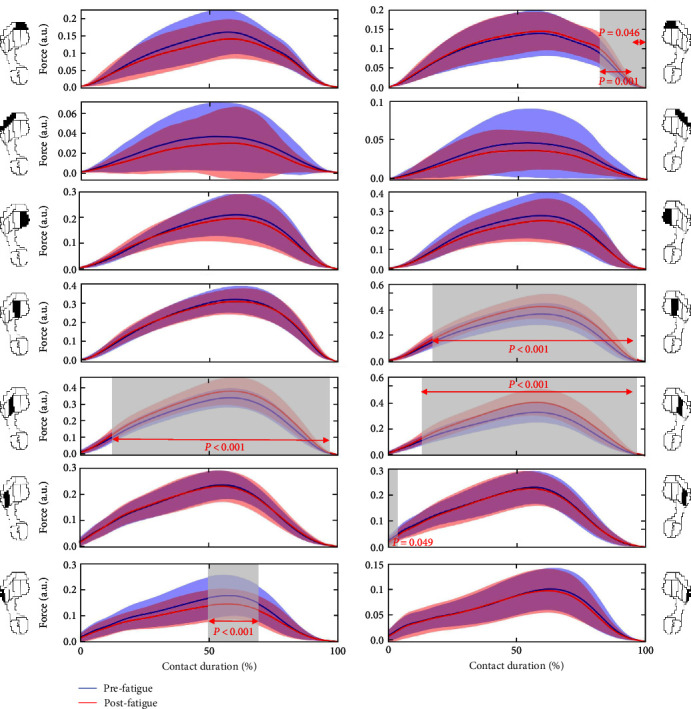
The time-series normalized force in the metatarsal areas in the pre-fatigue versus post-fatigue at the nondominant and dominant foot during running gait. *Note*. Nondominant side = left foot; dominant side = right foot.

As shown in Figure 4, starting from initial nondominant foot contact, the force progression in forefoot regions differed between pre- and post-fatigue states. Specifically, the force in M3 shows a significant increase at 12%–79% of contact duration after fatigue. However, the force of M5 has decreased at 50%–69% of contact duration after fatigue (*p* < 0.001). For the dominant foot, there was a significant increase of force at OT (83%–95% (*p*=0.001), 96%–100% (*p*=0.046) of contact duration), M2(17%–97%, *p* < 0.001), and M3(14%–97%, *p* < 0.001) after running-induced fatigue. However, the force of M4 decreased at 0%–3% of contact duration after fatigue (*p*=0.049).

### 3.2. Force Development in the Middle Foot, Heel, and Sum Areas

**Figure 5:**
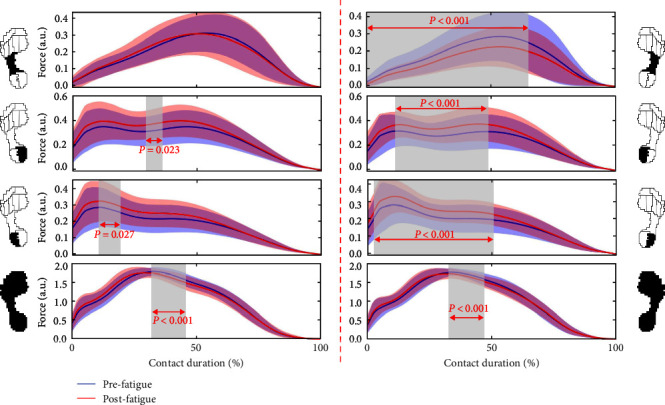
The time-series normalized force in the middle foot, heel, and sum areas in the pre-fatigue versus post-fatigue at the nondominant and dominant foot during running gait. *Note*. Nondominant side = left foot; dominant side = right foot.

As shown in Figure 5, there was no difference in MF at the nondominant foot. Interestingly, there was a significant decrease in plantar force at the dominant foot (0%–65%, *p* < 0.001). The heel regions were directly affected by running fatigue. For nondominant and dominant plantar forces, they were significantly increased at HM (nondominant: 30%–36%, *p*=0.023; dominant: 11%–49%, *p* < 0.001) and HL regions (nondominant: 11%–19%, *p*=0.027; dominant: 3%–51%, *p* < 0.001). However, the sum of forces from all ten regions at nondominant (33%–46%, *p* < 0.001) and dominant (34%–47%, *p* < 0.001) significantly decreased after running-induced fatigue.

### 3.3. Relative Time of Peak Force

As shown in Table 2, the relative time of peak force was significantly shortened at MF (*p*=0.001) and SUM (*p*=0.01) regions at nondominant feet in a fatigued state. Similarly, there was a significant shortening in the relative time of peak force at M5 of the dominant foot after fatigue. Interestingly, for H regions, the relative time of peak force was significantly delayed.

### 3.4. Representations of Deep Learning Models

**Figure 6:**
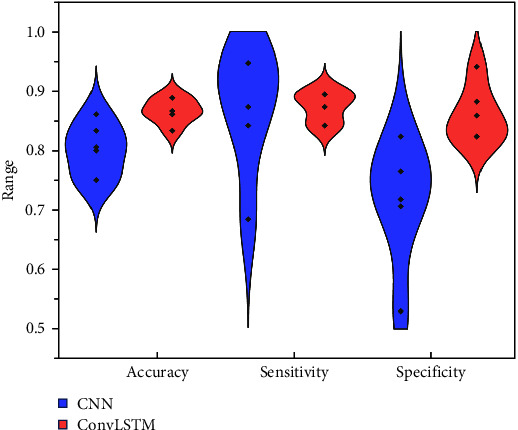
Violin plots of the classification results of total plantar pressure at CNN and ConvLSTM models.

**Figure 7:**
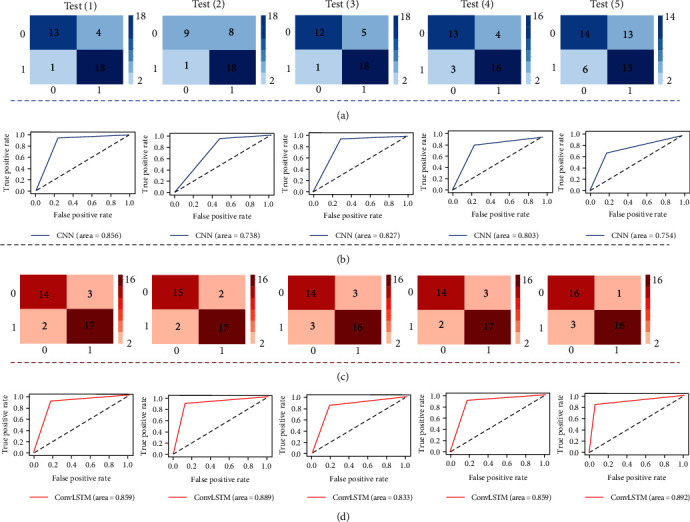
Confusion matrix and ROC of CNN and ConvLSTM models for 5 tests, respectively. (a) Confusion matrix of the CNN model; (b) ROC of the CNN model; (c) confusion matrix of the ConvLSTM model; (d) ROC of the ConvLSTM model.

The classification results of total plantar pressure at CNN and ConvLSTM model are shown in Figure 6, and the confusion matrix and ROC of each model are shown in Figure 7.  Table 3 presents the average accuracy, specificity, and sensitivity derived from the five test sets. The ConvLSTM model outperformed the CNN with an accuracy of 86.7% versus 80%. Likewise, ConvLSTM's specificity was superior at 85.9%, compared to CNN's 71.8%. Nonetheless, both models matched with a sensitivity rate of 87.4%.

## 4. Discussion

The aim of this investigation was to investigate the effect of running fatigue on the bilateral plantar force distribution of the foot and the effectiveness of CNN and ConvLSTM models for fatigue gait recognition. The results of this study showed that running fatigue changed the distribution pattern of load on the plantar of the dominant and nondominant limbs. These changes are similar to previous studies [6, 8, 11]. The force distribution of the dominant plantar of runners shown major differences reflected in reduced force under the midfoot at the expense of increased force under the H, M2, and M3. This increased loading of the medial forefoot region is in agreement with previously demonstrated higher pressures under the forefoot and lower peak pressures under the midfoot, which were reported by Bisiaux and Moretto [10] after fatigue induced by an intensive 30-min run. These results may indicate that the load was transferred from the midfoot to the toes and metatarsals [10]. The increased loading in M2–3 may be related to reduced activity of the toe flexors and posterior tibial muscles after running fatigue [31]. In addition, Arndt et al. [32] have also reported that higher strain rates and deformation of metatarsal bones can also occur after muscle fatigue caused by running. These findings could be a risk factor for a metatarsal stress fracture [10]. Especially the M2 and M3 are vulnerable because of the difference between the applied plantar pressure and bone strength [31].

Previous studies have demonstrated that dominant feet play a propulsive role, while nondominant feet are more likely to function as a stable gait [15]. Excessive force at the H region after fatigue appears in the dominant limb may be a compensatory effect of the functionally driven winch mechanism [5]. Willson and Kernozek [11] reported that running fatigue could cause changes in plantar surface loading characteristics and running technique. This study showed that the force of M5 at the nondominant foot has decreased at the metaphase (50%–69%) of contact duration, while the force of M3 has increased significantly at most of the contact duration (12%–79%) after fatigue, suggesting that the fatigue transferred foot loading from the lateral region toward the inside of the foot, especially in the nondominant foot [13]. This finding may be a weakening of the function of the nondominant limb to stabilize gait after muscle fatigue [21]. In addition, the relative time of peak force of MF at the nondominant was significantly shortened after fatigue, suggesting that more impulse was concentrated in the MF region. This finding may be due to the damage to the active control mechanism of the MF during the contact stage, leading to a reduction in the cushioning function of the nondominant plantar, which was the potential factors for plantar fasciitis [33]. Interestingly, the relative time of the peak of H-region force at the dominant foot was significantly delayed after fatigue. This finding may be a compensatory mechanism to maintain the propulsive function of the dominant limb, making the gravitational torque between the heel and toe region more even in the later stages of push-off [6].

Several reports have investigated the influence of the range of motion in the coronal plane of the foot on shock attenuation at heel strikes [34, 35]. In addition, the ability of the musculoskeletal system to attenuate the shock magnitude generated during heel strikes also decays with fatigue [36]. In our study, the plantar forces recorded under the HM and HL regions of both dominant and nondominant revealed the changes which running fatigue during the loading stage. Without other direct measurements, we can only speculate that excessive heel loading after fatigue may be linked to weaker muscle strength, which controls the movement of the ankle joint in the coronal plane after fatigue [10]. These observations were consistent with several previous studies [2, 7]. Interestingly, the sum of forces from all ten regions at nondominant of 33%–46% of contact duration significantly decreased, and the relative time of peak force was significantly shortened after running-induced fatigue, suggesting that dorsiflexor fatigue led to more vertical loading rate on the plantar. A significant interaction between loading rate and running-related calf, foot, and ankle injuries was demonstrated in a study by Gerlach et al. [37]. The results of this study could provide necessary enlightenment about the condition of different running-related injuries among runners with limbs on different sides.

In addition, by applying the feature set of the time series bilateral plantar force data to specific deep learning predictive models for running fatigue gait, the results showed that both CNN and ConvLSTM models have good performance in predicting fatigue gait automatically. As expected, the ConvLSTM model (85.9%, 88.9%, 83.3%, 85.9%, and 89.2%, respectively) has better accuracy compared to the CNN model (85.6%, 73.8%, 82.7%, 80.3%, and 75.4%) in all five tests, suggesting that ConvLSTM performs better for multi-feature data with simultaneous spatiotemporal dependence [27]. Traditional time series biomechanical datasets are all characterized by high dimensionality, high variability, time dependence, and nonlinearity [38]. Therefore, with the promising findings from this study as a foundation, future research suggests applying the Convlstm model to other analyses, such as marker trajectories, ground reaction forces, myoelectric signals, and other prediction and classification needs. In addition, as shown in Table 3, the specificity of the ConvLSTM model was also higher than that of the CNN, indicating that it could detect fatigue gait better, while the performance of sensitivity was consistent in both models, indicating that both models were equally effective in predicting non-fatigue gait.

There are four limitations to this study. Possible differences in plantar pressure distribution patterns between overground conditions and treadmill conditions were reported by García-Pérez et al. [39]. In this study, the running-induced fatigue protocol was carried out on a treadmill. Thus, further investigation is needed to support our findings in overground conditions. The runners were evaluated under barefoot conditions, potentially overlooking the impact of footwear on post-fatigue running posture [40]. Additionally, we selected only two deep learning models (CNN and ConvLSTM) for data training based on data features, and more comparisons of classifiers (such as deep neural network) for plantar pressure feature discovery should be developed in future studies. At the end, only the pedobarographic data of amateur male runners was included in this study; whether the model developed in this study applies to female or elite runners should be verified in future studies.

## 5. Conclusions

A running-induced fatigue protocol caused different changes in the distribution of plantar force on the dominant and nondominant limbs. These changes may be part of the underlying mechanism of unilateral limb overuse injuries. Future discussions of lower limb lesions or running-related injuries should take this into account. Furthermore, the ConvLSTM model showed high performance (acc = 0.867) in detecting fatigue gait, and it outperformed the CNN model (0.800). This will broaden the possibilities for future research on running-related gait biomechanical feature recognition and enhance the development of fatigue monitoring tools.

## Figures and Tables

**Table 1:** Anthropometric characteristics of the recruited participants.

Information	Mean	S.D.
Age (year)	24.27	1.36
Height (cm)	177.00	4.33
Weight (kg)	69.80	8.46
BMI (kg/m^2^)	22.20	1.7

**Table 2:** The relative time of peak force in the 10 areas in the pre-fatigue versus post-fatigue at the nondominant and dominant foot during running gait.

Areas	Non-dominant foot			Dominant foot		
	Pre (%)	Post (%)	*p*-Value	Pre (%)	Post (%)	*p*-Value
H	56.99 (9.07)	57.26 (11.14)	0.84	55.6 (12.71)	58.9 (13.02)	**0.04** ^*∗*^
OT	52.51 (11.90)	55 (13.46)	0.17	53.34 (11.59)	52.43 (12.95)	0.58
M1	56.99 (9.33)	55.8 (11.58)	0.27	56.22 (10.75)	56.33 (10.62)	0.92
M2	58.97 (6.19)	58.7 (7.09)	0.74	58.47 (5.96)	58.32 (6.38)	0.86
M3	56.74 (3.46)	56.87 (4.62)	0.22	56.98 (4.39)	56.51 (5.23)	0.49
M4	54.46 (5.01)	54.00 (7.09)	0.56	55.93 (5.60)	55.70 (6.31)	0.77
M5	57.03 (7.88)	57.38 (9.23)	0.76	62.23 (6.59)	59.62 (11.30)	**0.05** ^*∗*^
MF	53.26 (6.40)	50.50 (7.14)	**0.001** ^*∗*^	53.87 (6.75)	53.61 (8.09)	0.81
HM	25.37 (18.94)	25.3 (17.58)	0.97	24.48 (19.59)	24.57 (17.30)	0.97
HL	11.02 (11.54)	11.62 (10.25)	0.72	14.50 (15.19)	11.29 (10.64)	0.07
SUM	31.61 (4.76)	29.7 (6.06)	**0.01** ^*∗*^	33.47 (6.32)	32.38 (7.86)	0.28

*Note*. “ ^*∗*^” means significant difference between pre- and post-fatigue state (*p* ≤ 0.05). Nondominant foot = left foot; dominant foot = right foot.

**Table 3:** Classification metrics of total plantar pressure by two models.

Model	Accuracy	Sensitivity	Specificity
CNN	0.800	0.874	0.718
ConvLSTM	0.867	0.874	0.859

## Data Availability

Data are available upon request due to privacy restrictions. The data presented in this study may be available upon request from the corresponding author and with the authorization of funding origination.

## References

[1] Buist I., Bredeweg S. W., Lemmink K. A. P. M., van Mechelen W., Diercks R. L. (2010). Predictors of running-related injuries in novice runners enrolled in a systematic training program: a prospective cohort study. *The American Journal of Sports Medicine*.

[2] Willems T. M., De Ridder R., Roosen P. (2012). The effect of a long-distance run on plantar pressure distribution during running. *Gait & Posture*.

[3] Meeuwisse W. H. (1994). Assessing causation in sport injury: a multifactorial model. *Clinical Journal of Sport Medicine*.

[4] Fourchet F., Kelly L., Horobeanu C., Loepelt H., Taiar R., Millet G. (2015). High-intensity running and plantar-flexor fatigability and plantar-pressure distribution in adolescent runners. *Journal of Athletic Training*.

[5] Gao Z., Mei Q., Xiang L., Gu Y. (2020). Difference of walking plantar loadings in experienced and novice long-distance runners. *Acta of Bioengineering and Biomechanics*.

[6] Zhang X., Wang W., Chen G., Ji A., Song Y. (2021). Effects of standing and walking on plantar pressure distribution in recreational runners before and after long-distance running. *Journal of Biomechanics*.

[7] Willems T. M., De Clercq D., Delbaere K., Vanderstraeten G., De Cock A., Witvrouw E. (2006). A prospective study of gait related risk factors for exercise-related lower leg pain. *Gait & Posture*.

[8] Nagel A., Fernholz F., Kibele C., Rosenbaum D. (2008). Long distance running increases plantar pressures beneath the metatarsal heads: a barefoot walking investigation of 200 marathon runners. *Gait & Posture*.

[9] Weist R., Eils E., Rosenbaum D. (2004). The influence of muscle fatigue on electromyogram and plantar pressure patterns as an explanation for the incidence of metatarsal stress fractures. *The American Journal of Sports Medicine*.

[10] Bisiaux M., Moretto P. (2008). The effects of fatigue on plantar pressure distribution in walking. *Gait & Posture*.

[11] Willson J. D., Kernozek T. W. (1999). Plantar loading and cadence alterations with fatigue. *Medicine and Science in Sports and Exercise*.

[12] Hesar N. G. Z., Van Ginckel A., Cools A. (2009). A prospective study on gait-related intrinsic risk factors for lower leg overuse injuries. *British Journal of Sports Medicine*.

[13] Anbarian M., Esmaeili H. (2016). Effects of running-induced fatigue on plantar pressure distribution in novice runners with different foot types. *Gait & Posture*.

[14] Hodges S. J., Patrick R. J., Reiser R. F. (2011). Effects of fatigue on bilateral ground reaction force asymmetries during the squat exercise. *The Journal of Strength & Conditioning Research*.

[15] Gao Z., Mei Q., Xiang L., Baker J. S., Fernandez J., Gu Y. (2022). Effects of limb dominance on the symmetrical distribution of plantar loading during walking and running. *Proceedings of the Institution of Mechanical Engineers, Part P: Journal of Sports Engineering and Technology*.

[16] Dong H., Ugalde I., Figueroa N., Saddik A. E. (2014). Towards whole body fatigue assessment of human movement: a fatigue-tracking system based on combined semg and accelerometer signals. *Sensors*.

[17] Jiang Y., Hernandez V., Venture G., Kulić D., Chen K., B (2021). A data-driven approach to predict fatigue in exercise based on motion data from wearable sensors or force plate. *Sensors*.

[18] Xiang L., Gu Y., Mei Q., Wang A., Shim V., Fernandez J. (2022). Automatic classification of barefoot and shod populations based on the foot metrics and plantar pressure patterns. *Frontiers in Bioengineering and Biotechnology*.

[19] Xiang L., Wang A., Gu Y., Zhao L., Shim V., Fernandez J. (2022). Recent machine learning progress in lower limb running biomechanics with wearable technology: a systematic review. *Frontiers in Neurorobotics*.

[20] Mei Q., Gu Y., Xiang L. (2020). Foot shape and plantar pressure relationships in shod and barefoot populations. *Biomechanics and Modeling in Mechanobiology*.

[21] Gao Z., Fekete G., Baker J. S., Liang M., Xuan R., Gu Y. (2022). Effects of running fatigue on lower extremity symmetry among amateur runners: from a biomechanical perspective. *Frontiers in Physiology*.

[22] Cifrek M., Medved V., Tonković S., Ostojić S. (2009). Surface EMG based muscle fatigue evaluation in biomechanics. *Clinical Biomechanics*.

[23] Liang S., Liu Y., Li G., Zhao G. Elderly fall risk prediction with plantar center of force using convlstm algorithm.

[24] Song Y., Cen X., Chen H. (2023). The influence of running shoe with different carbon-fiber plate designs on internal foot mechanics: a pilot computational analysis. *The Journal of Biomechanics*.

[25] Wen J., Ding Q., Yu Z., Sun W., Wang Q., Wei K. (2012). Adaptive changes of foot pressure in hallux valgus patients. *Gait & Posture*.

[26] Sherstinsky A. (2020). Fundamentals of recurrent neural network (RNN) and long short-term memory (LSTM) network. *Physica D: Nonlinear Phenomena*.

[27] Shi X., Chen Z., Wang H., Yeung D.-Y., Wong W.-K., Woo W. C. (2015). Convolutional LSTM network: a machine learning approach for precipitation nowcasting. *Advances in Neural Information Processing Systems*.

[28] Koblbauer I. F., van Schooten K. S., Verhagen E. A., van Dieën J. H. (2014). Kinematic changes during running-induced fatigue and relations with core endurance in novice runners. *Journal of Science and Medicine in Sport*.

[29] Gao Z., Mei Q., Fekete G., Baker J. S., Gu Y. (2020). The effect of prolonged running on the symmetry of biomechanical variables of the lower limb joints. *Symmetry*.

[30] Borg G. (1998). *Borg’s Perceived Exertion and Pain Scales*.

[31] Griffin N. L., Richmond B. G. (2005). Cross-sectional geometry of the human forefoot. *Bone*.

[32] Arndt A., Ekenman I., Westblad P., Lundberg A. (2002). Effects of fatigue and load variation on metatarsal deformation measured in vivo during barefoot walking. *Journal of Biomechanics*.

[33] Shiotani H., Mizokuchi T., Yamashita R., Naito M., Kawakami Y. (2020). Acute effects of long-distance running on mechanical and morphological properties of the human plantar fascia. *Scandinavian Journal of Medicine & Science in Sports*.

[34] Perry S. D., Lafortune M. A. (1995). Influences of inversion/eversion of the foot upon impact loading during locomotion. *Clinical Biomechanics*.

[35] Vijayvargiya A., Dhanka B., Gupta V., Kumar R. (2023). Implementation of machine learning algorithms for automated human gait activity recognition using sEMG signals. *International Journal of Biomedical Engineering and Technology*.

[36] Dempster J., Dutheil F., Ugbolue U. C. (2021). The prevalence of lower extremity injuries in running and associated risk factors: a systematic review. *Physical Activity and Health*.

[37] Gerlach K. E., White S. C., Burton H. W., Dorn J. M., Leddy J. J., Horvath P. J. (2005). Kinetic changes with fatigue and relationship to injury in female runners. *Medicine and Science in Sports and Exercise*.

[38] Wang S. (2022). Pattern-matching kinematic analysis of glide phase after start with different techniques in medley swimming: an olympic champion case. *Physical Activity and Health*.

[39] García-Pérez J. A., Pérez-Soriano P., Llana S., Martínez-Nova A., Sánchez-Zuriaga D. (2013). Effect of overground vs treadmill running on plantar pressure: influence of fatigue. *Gait & Posture*.

[40] Xiang L., Gu Y., Wang A., Shim V., Gao Z., Fernandez J. (2023). Foot pronation prediction with inertial sensors during running: a preliminary application of data-driven approaches. *Journal of Human Kinetics*.

